# First-trimester thyroid reference intervals in twin pregnancy: establishment, diagnostic utility, and association with adverse outcomes

**DOI:** 10.3389/fendo.2025.1700641

**Published:** 2025-11-28

**Authors:** Zhangli Dong, Jianxia Lin, Mi Han, Yu Meng

**Affiliations:** 1 Department of Obstetrics and Gynecology, International Peace Maternity and Child Health Hospital, School of Medicine, Shanghai Jiao Tong University, Shanghai, China; 2 Shanghai Key Laboratory of Embryo Original Diseases, Shanghai, China

**Keywords:** pregnancy outcomes, twin, thyroid function, free thyroxine, thyroid-stimulating hormone

## Abstract

**Objective:**

This large retrospective study aims to establish twin pregnancy-specific reference intervals for serum TSH and FT4 in early gestation and evaluate their clinical utility.

**Methods:**

This study analyzed TSH and FT4 levels in early pregnancy among 46,474 singleton and 1,183 twin pregnancies at a tertiary hospital. Local gestation-specific reference intervals for TSH and FT4 were established by calculating the 2.5th to 97.5th percentiles. Chi-square tests were used to compare abnormal detection rates, univariate regression analyzed differences in thyroid dysfunction prevalence, and multivariate regression assessed their predictive value for adverse pregnancy outcomes. Stratified, interaction, and marginal analyses were performed to evaluate the consistency and clinical relevance of the association.

**Results:**

Twin pregnancies showed lower median TSH (0.62 vs. 1.17 mIU/L) and higher FT4 (15.40 vs. 14.80 pmol/L) than singletons. Twin-specific intervals can reduce abnormal TSH/FT4 detection (P<0.001). Based on the twin-specific intervals, low TSH was significantly associated with preeclampsia, whereas low FT4 was linked to SGA, LGA, emergency cesarean delivery, and fetal distress (P<0.05). Multivariable logistic regression confirmed that reduced FT4 independently predicted risks of SGA and LGA. Interaction analyses showed the FT4–growth abnormality association was consistent across different maternal characteristics. Marginal value analysis showed that FT4 levels between P2.5 and P5 did not significantly increase risks of SGA or LGA, supporting the clinical validity of the current reference range.

**Conclusion:**

Twin-specific thyroid reference intervals in early pregnancy can improve the accuracy of high-risk pregnancy identification and show strong potential for clinical application.

## Introduction

Pregnancy involves significant physiological and hormonal changes, which can complicate the accurate measurement and interpretation of thyroid function. Twin pregnancies, in particular, present unique challenges due to their distinct physiological adaptations, and they are associated with a higher risk of pregnancy-related complications and adverse outcomes. Thyroid dysfunction has been widely recognized as closely associated with various pregnancy outcomes, including preterm birth, preeclampsia, and fetal growth abnormalities (1). However, the thyroid function reference ranges currently used in clinical practice are mostly derived from singleton pregnancies, lacking specific standards tailored for twin gestations.

It is widely recommended by various international guidelines to employ region- and laboratory-specific reference ranges for the assessment of thyroid function in pregnancy, thereby enhancing diagnostic accuracy and clinical utility. Considering physiological differences among populations, such as fetal number and gestational age, developing region- and gestational age-specific reference standards is equally important (2). Serum human chorionic gonadotropin (hCG) levels are higher in twin pregnancies than in singletons. Since hCG stimulates the thyroid to produce more free thyroxine (FT4), potentially leading to suppressed thyroid-stimulating hormone (TSH) levels, reference ranges for thyroid-related markers in early pregnancy may differ between singleton and twin gestations (3, 4). Applying singleton reference standards directly to twins may result in misdiagnosis or overdiagnosis of thyroid dysfunction, adversely affecting subsequent risk screening and intervention decisions. Therefore, establishing thyroid function reference intervals tailored to the physiological characteristics and regional features of twin pregnancies, and exploring their associations with adverse pregnancy outcomes, holds significant clinical and public health value for optimizing twin pregnancy management and improving maternal and neonatal health.

Currently established thyroid reference intervals during pregnancy are primarily based on singleton pregnancies, while research on gestation-specific reference ranges for twin pregnancies remains limited—particularly among Chinese populations. This study aims to establish early pregnancy twin-specific reference intervals for TSH and FT4 using a large cohort from our hospital. We compared the twin-specific reference intervals with those from singleton pregnancies and ATA-recommended ranges to assess their effectiveness in detecting thyroid dysfunction and predicting adverse pregnancy outcomes. The goal is to provide evidence-based support for the early identification and precision management of thyroid dysfunction in twin pregnancies.

## Methods

### Study design and population

**Figure 1:**
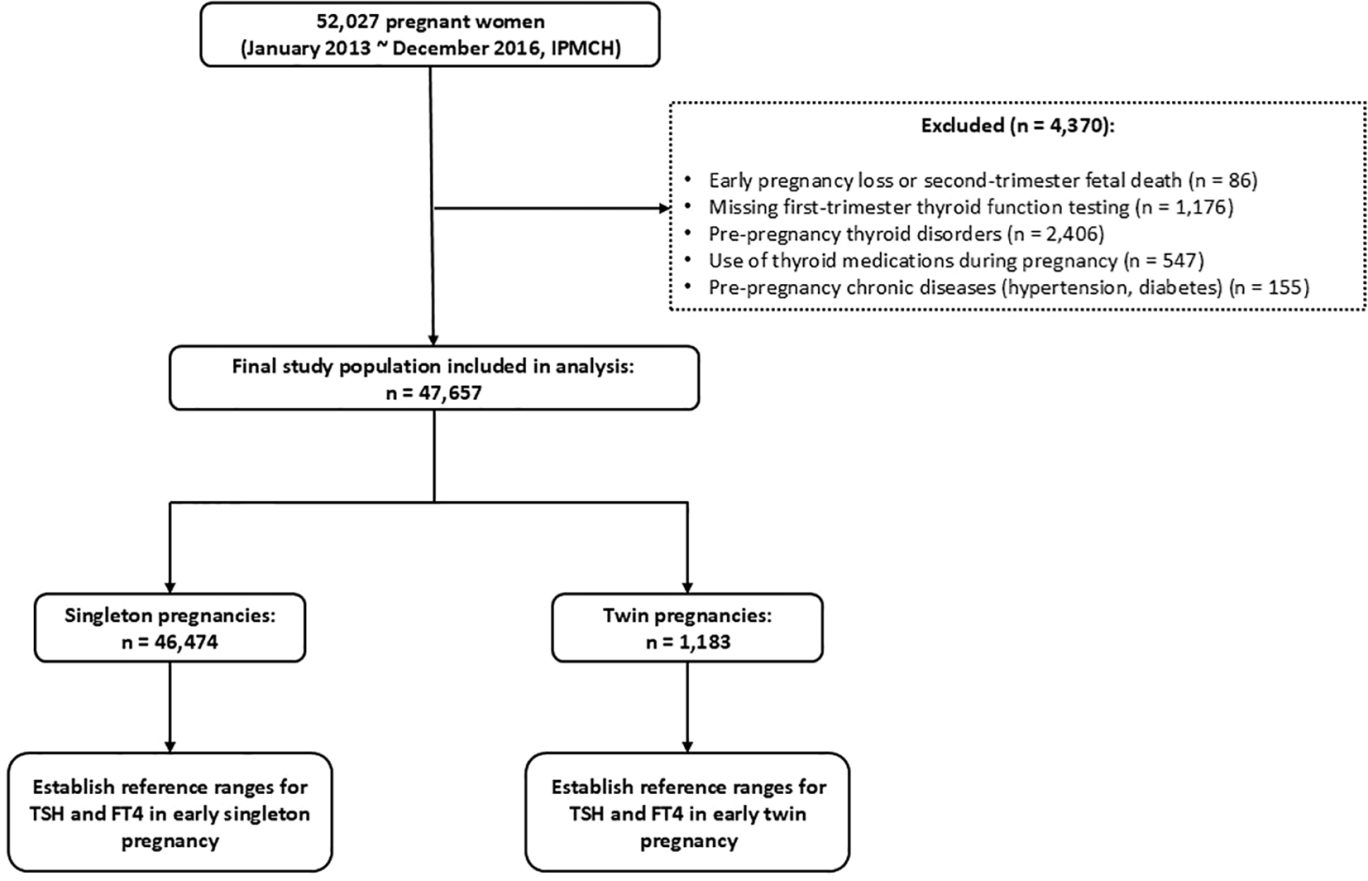
Flowchart of participant inclusion and exclusion.

This retrospective cohort study included data from women with singleton and twin pregnancies who received prenatal care at the International Peace Maternity and Child Health Hospital (IPMCH), a tertiary specialized hospital in Shanghai, between January 2013 and December 2016. A total of 52,027 pregnant women were initially enrolled. After excluding 86 cases of early miscarriage or mid-trimester fetal loss, 1,176 cases without thyroid function testing in early pregnancy, 2,406 women with pre-existing thyroid disorders, 547 women who used thyroid medications during pregnancy, and 155 women with pre-existing chronic conditions (hypertension or diabetes), a final cohort of 47,657 women was included. Among them, 46,474 had singleton pregnancies and 1,183 had twin pregnancies. The detailed inclusion and exclusion process is shown in **Figure 1**.

### Data collection

This retrospective cohort study extracted basic maternal information—including age, gravidity, parity, pre-pregnancy body mass index (BMI), and education level—from the hospital’s electronic medical record system and related databases. Serum thyroid function indicators such as TSH, FT4, and thyroid peroxidase antibody (TPOAb) were collected from early pregnancy (11–13 weeks gestation) laboratory results. Pregnancy outcome data were retrospectively compiled, including early preterm birth (<34 weeks), large for gestational age (LGA), small for gestational age (SGA), fetal distress, emergency cesarean delivery, as well as pregnancy-related complications such as gestational diabetes mellitus (GDM) and preeclampsia.

### Thyroid function testing

In this study, fasting serum samples were collected from participants via median cubital vein puncture during early pregnancy (11–13 weeks gestation). Samples were centrifuged within 6 hours of collection to separate serum. According to the reagent manufacturer’s instructions, concentrations of TSH, FT4, and TPOAb were measured using the Architect i2000 immunoassay analyzer (Abbott Laboratories, Chicago, USA). The intra-assay and inter-assay coefficients of variation (CVs) were 3.10% and 3.50% for TSH, 3.80% and 5.70% for FT4, respectively. For TPOAb, the total CV ranged from 1.31% to 10.00% according to manufacturer specifications and published multicenter evaluations, consistent with the assay’s performance characteristics. Reference intervals for TSH and FT4 were defined as the 2.5th to 97.5th percentiles of the general population. Per the manufacturer’s guidelines, a TPOAb level ≥ 5.61 IU/mL was considered positive.

### Establishment of reference intervals and definitions of thyroid disorders

After excluding subjects who met the exclusion criteria, this study ultimately included 46,474 singleton and 1,183 twin pregnancies. Early pregnancy thyroid function data were extracted for analysis. The median, 2.5th percentile (P2.5), and 97.5th percentile (P97.5) of TSH and FT4 were calculated to establish regional reference intervals for thyroid function in both singleton and twin pregnancies. Based on the established twin-specific reference intervals, thyroid dysfunction subtypes were defined as follows: overt hyperthyroidism was defined as TSH < P2.5 and FT4 > P97.5; subclinical hyperthyroidism as TSH < P2.5 and FT4 within the reference range; overt hypothyroidism as TSH > P97.5 and FT4 < P2.5; subclinical hypothyroidism as TSH > P97.5 and FT4 within the reference range; and isolated hypothyroxinemia as FT4 < P2.5 and TSH within the reference range.

### Definition of adverse pregnancy outcomes

In this study, the definitions of adverse pregnancy outcomes are based on medical guidelines and literature reports, as detailed below: (I) Preeclampsia (PE): According to the American College of Obstetricians and Gynecologists (ACOG) guideline (5), preeclampsia is defined as new-onset hypertension after 20 weeks of gestation in women with previously normal blood pressure, with systolic blood pressure ≥140 mmHg or diastolic blood pressure ≥90 mmHg measured at least twice, 4 hours apart, accompanied by one or more of the following: proteinuria with 24-hour urinary protein excretion ≥300 mg or a protein-to-creatinine ratio ≥0.325. (II) SGA and LGA: In clinical practice, singleton fetal growth standards are commonly used to assess the growth of twin fetuses; however, this may lead to misclassification of many twins as SGA or LGA, affecting clinical judgment and intervention strategies (6). Moreover, fetal weight and growth patterns differ significantly among various ethnicities and populations. Therefore, this study applied the twin birth weight reference standards developed by Zhang et al. based on the Chinese population (7) to improve assessment accuracy. In this study, birth weight below the 10th percentile (P10) for the same gestational age was defined as SGA, and birth weight above the 90th percentile (P90) was defined as LGA. (III) Early Preterm Birth: Studies report that approximately 60% of twin pregnancies deliver before 37 weeks of gestation (8), indicating a much higher rate of preterm birth in twins compared to singletons. To more accurately reflect the pregnancy outcomes specific to twins, this study defined early preterm birth as delivery before 34 weeks of gestation. (IV) GDM: Diagnosis was based on a 75-g oral glucose tolerance test (OGTT) performed between 24 and 28 weeks of gestation. The diagnostic criteria included any of the following: fasting plasma glucose ≥ 5.1 mmol/L, 1-hour plasma glucose ≥ 10.0 mmol/L, or 2-hour plasma glucose ≥ 8.5 mmol/L. (V) Fetal Distress: Defined as signs of intrauterine hypoxia or acidosis, indicated by abnormal fetal heart rate monitoring, umbilical artery blood gas pH <7.2, meconium-stained amniotic fluid, or a 1-minute Apgar score <7.

### Statistical analysis

Descriptive statistics were used to analyze the baseline characteristics of the pregnant women and thyroid function parameters. Continuous variables were presented as mean ± standard deviation (Mean ± SD) or median with interquartile range (Median, IQR), while categorical variables were expressed as frequency and percentage (n, %). The reference ranges for serum TSH and FT4 levels in early pregnancy were established using a non-parametric method to calculate the 2.5th (P2.5) and 97.5th percentiles (P97.5) in twin pregnancies. The same approach was applied to data from singleton pregnancies during the corresponding gestational period. Comparisons of reference intervals between twin and singleton groups were conducted using the Mann–Whitney U test (for non-normally distributed data) or the t-test (for normally distributed data). Based on the established reference ranges, participants were categorized into normal and abnormal thyroid function groups. Univariate analysis (χ² test or Fisher’s exact test) was performed to compare the incidence of adverse pregnancy outcomes and complications between groups. Multivariate logistic regression analysis was then used to adjust for potential confounding factors, including maternal age, parity, and BMI, to assess the association between thyroid dysfunction and adverse pregnancy outcomes (e.g., preterm birth, macrosomia, fetal distress). The results were reported as adjusted odds ratios (ORs) with 95% confidence intervals (CIs). All statistical analyses were performed using SPSS version 25.0, and a two-sided P-value <0.05 was considered statistically significant.

## Results

### Baseline characteristics of singleton and twin pregnancies

Based on the inclusion and exclusion criteria of this study, a total of 47,657 pregnant women were enrolled, including 1,183 singleton pregnancies and 46,474 twin pregnancies. The baseline characteristics of the two groups are presented in **Table 1**. Given the retrospective observational design, statistical comparisons for baseline characteristics were performed using the Chi-square test for categorical variables and the Mann-Whitney U test for continuous variables, and P values are provided to indicate potential differences between singleton and twin pregnancies. As shown in **Table 1**, maternal age, pre-pregnancy BMI, education level, and IVF conception differed significantly between the two group. Therefore, these variables were adjusted as confounders in the subsequent multivariate analyses.

**Table 1:** Baseline characteristics of pregnant women with singleton and twin pregnancies.

Baseline characteristics		Twin (n = 1,183)	Singleton (n = 46,474)	P
Maternal Age (years), Median [Q1, Q3]		31 [29, 34]	30 [28, 32]	<0.001
Pre-pregnancy BMI (kg/m^2^), Median [Q1, Q3]		21.1 [19.5, 23.4]	20.6 [19.1, 22.5]	<0.001
Education Levels, n (%)				<0.001
	High school or below	387 (32.9%)	10,886 (23.4%)	
	Bachelor’s degree or above	796 (67.1%)	35,588 (76.6%)	
TPOAb Positive, n (%)		106 (9.0%)	4,717 (10.1%)	0.180
IVF conception, n (%)		643 (54.4%)	1,400 (3.0%)	<0.001

Data are presented as median [Q1, Q3] for continuous variables and number (percentage) for categorical variables. Percentages are calculated based on the total number of participants in each group (Singleton: n = 46,474; Twin: n =1,183). Minor discrepancies may exist due to rounding. BMI, body mass index; TPOAb, thyroid peroxidase antibody; IVF, *in vitro* fertilization; Q1, first quartile; Q3, third quartile.

### Distribution characteristics of TSH and FT4 levels in women with twin pregnancies in the first trimester

**Figure 2:**
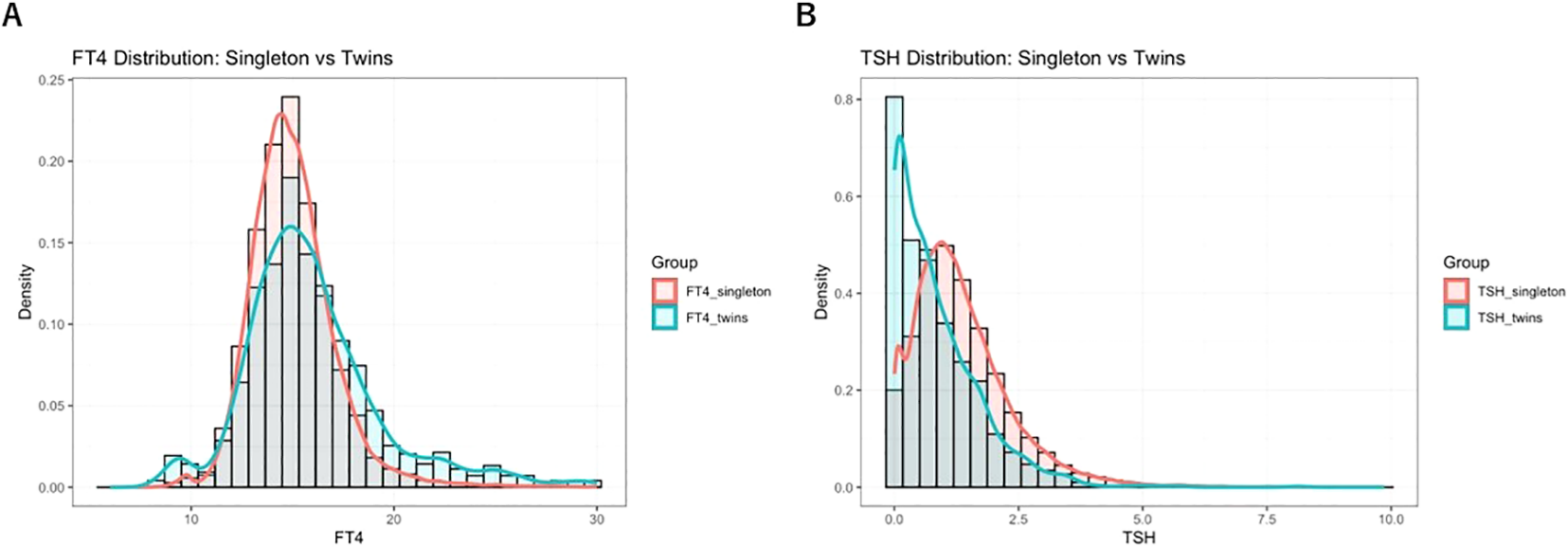
Distribution of TSH and FT4 levels in twin pregnancies during early pregnancy and comparison with singleton pregnancy reference intervals: Panel **(A)** shows the distribution of FT4 levels; Panel **(B)** shows TSH levels. The figure illustrates the distribution of early pregnancy TSH and FT4 in 1,183 twin pregnancies and 46,474 singleton pregnancies, presented as density curves and histograms. The green shaded area represents the locally established 95% reference interval (P2.5–P97.5) for twin pregnancies, while the red shaded area indicates the corresponding reference interval for singleton pregnancies. Notable differences in the distributions of TSH and FT4 levels were observed between twin and singleton pregnancies, highlighting the distinct thyroid function profiles associated with multiple gestations.

This study collected early pregnancy thyroid function data from singleton and twin pregnancies at our institution, specifically measuring serum levels of TSH and FT4. The reference ranges for TSH and FT4 in different pregnancy types were described using medians and 95% reference intervals, based on data from the local population. The aim was to compare thyroid function characteristics between singleton and twin pregnancies. In singleton pregnancies, considered the reference group, the median TSH level in early pregnancy was 1.17 mIU/L, with a 95% reference interval of 0.03–3.64 mIU/L. The median FT4 level was 14.80 pmol/L, with a reference interval of 11.70–19.60 pmol/L. In contrast, twin pregnancies showed markedly different thyroid function profiles. The median TSH level was significantly lower at 0.62 mIU/L (95% reference interval: 0.01–2.99 mIU/L), while the median FT4 level was significantly higher at 15.40 pmol/L (95% reference interval: 9.76–24.95 pmol/L), as shown in **Figure 2**. The comparison between singleton and twin pregnancies revealed significant differences in thyroid function characteristics. These findings highlight the distinct thyroid function profile in twin pregnancies, emphasizing the need for pregnancy-specific reference intervals.

### Comparison of abnormal TSH and FT4 prevalence under different reference ranges

**Figure 3:**
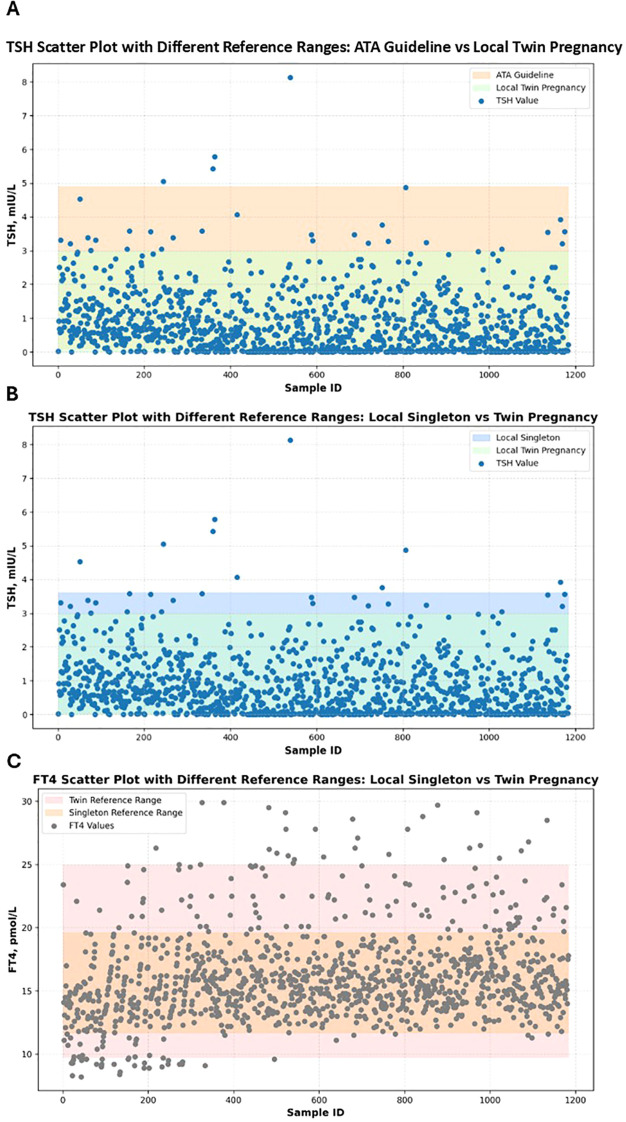
Scatter plots of serum TSH and FT4 levels in twin pregnancies according to different reference intervals. Panel **(A)** shows TSH scatter plot with different reference ranges: ATA guideline vs local twin pregnancy. Panel **(B)** shows TSH scatter plot with different reference ranges: local singleton vs twin pregnancy. Panel **(C)** shows FT4 scatter plot with different reference ranges: local singleton vs twin pregnancy. Scatter plots show the distribution of serum TSH and FT4 levels according to three diagnostic standards: the ATA guideline, local singleton-based reference ranges, and locally established twin-specific reference ranges. Colored shaded bands represent the normal reference ranges defined by different diagnostic standards. Each dot represents an individual measurement; deep blue dots denote TSH values, while dark gray dots denote FT4 values.

This study compared thyroid function abnormalities in twin pregnancies using three different reference ranges to evaluate the applicability and impact of twin-specific reference intervals. The first was the singleton pregnancy reference range recommended by the 2017 ATA guideline (9) which defines a normal TSH range of 0.1 ~ 4.0 mIU/L and aligns with the TSH reference range specified in China’s 2019 guidelines for the diagnosis and treatment of thyroid disease during pregnancy and postpartum (10). The second was a local singleton pregnancy reference range established from the current study cohort, where FT4 and TSH reference intervals were defined by the 2.5th and 97.5th percentiles as lower and upper limits, respectively. The local twin pregnancy–specific reference range derived from the distribution of FT4 and TSH levels in twin pregnancies within the cohort, using the 2.5th and 97.5th percentiles as cutoff values. Using these different reference ranges, abnormal TSH and FT4 levels (including both elevated and decreased values) were identified. The numbers and proportions of pregnant women classified as having thyroid dysfunction under each standard were calculated. Differences in abnormality rates between reference standards were analyzed using the Chi-square test to assess the impact of applying twin-specific reference intervals. The results are presented in **Table 2**. The results showed that when using the ATA guideline reference range, the prevalence of abnormal TSH was 24.4%. However, this rate significantly decreased to 5.1% when applying the twin-specific reference intervals (P < 0.001). Compared with the local singleton pregnancy reference ranges, the use of twin-specific reference intervals also significantly reduced the detection rates of abnormal TSH and FT4 levels, from 16.1% to 5.1% for both hormones (P < 0.001). To visualize the differences in thyroid function assessment among the three reference standards, scatter plots of TSH and FT4 levels were generated (**Figure 3**), illustrating how the use of different reference intervals may influence the diagnosis of thyroid dysfunction. These findings suggest that applying reference intervals not specific to twin pregnancies may lead to an overestimation of thyroid dysfunction prevalence.

**Table 2:** Comparison of abnormal TSH and FT4 prevalence under different reference ranges.

Indicator	Reference standard	Reference interval	Abnormal cases (n, %)	Relative reduction (%)	P
TSH	ATA Guideline	0.1–4.0 mIU/L	289 (24.4%)	Reference	<0.001*
	Local Twin Pregnancy	0.01–2.99 mIU/L	60 (5.1%)	–19.3	
TSH	Local Singleton	0.03–3.64 mIU/L	191 (16.1%)	Reference	<0.001*
	Local Twin Pregnancy	0.01–2.99 mIU/L	60 (5.1%)	–11.0	
FT4	Local Singleton	11.70–19.60 pmol/L	190 (16.1%)	Reference	<0.001*
	Local Twin Pregnancy	9.76–24.95 pmol/L	60 (5.1%)	–11.0	

Abnormal cases are presented as number and percentage (n, %). Relative reduction (%) indicates the decrease in abnormality rate when applying the local twin pregnancy–specific reference interval compared to the corresponding singleton or ATA guideline reference intervals. P-values were calculated using the Chi-square test. TSH, thyroid-stimulating hormone; FT4, free thyroxine.

### Comparison of thyroid dysfunction assessment results using singleton and twin reference ranges

We also applied thyroid function reference ranges specific to twin and singleton pregnancies to compare the incidence of various thyroid disorders between the two groups. The results showed no significant differences in the prevalence of any thyroid dysfunction between women with twin pregnancies and those with singleton pregnancies (**Table 3**). Specifically, the incidence of overt hyperthyroidism was slightly lower in the twin pregnancy group compared to the singleton group (0.93% vs. 1.20%), with no statistically significant difference (aOR = 0.41, 95% CI 0.15–1.18, P = 0.099). Overt hypothyroidism was not observed in the twin group (0%), while it was 0.14% in the singleton group (P = 0.982). The incidences of subclinical hyperthyroidism (1.61% vs. 1.29%, aOR = 1.03, 95% CI 0.53–2.00, P = 0.934), subclinical hypothyroidism (2.54% vs. 2.36%, aOR = 1.24, 95% CI 0.76–2.01, P = 0.394), and hypothyroxinemia (2.54% vs. 2.18%, aOR = 1.02, 95% CI 0.68–1.55, P = 0.910) were slightly higher in the twin group, but none of these differences reached statistical significance.

**Table 3:** Incidence of thyroid disorders in twin versus singleton pregnancies.

Disease type	Twin pregnancy (n=1,183)	Singleton pregnancy (n=46,474)	Adjusted odds ratios (aOR)	95% CI	P
Overt Hyperthyroidism	11 (0.93%)	557 (1.20%)	0.41	0.15–1.18	0.099
Overt Hypothyroidism	0 (0.00%)	63 (0.14%)	—	—	0.982
Subclinical Hyperthyroidism	19 (1.61%)	602 (1.29%)	1.03	0.53–2.00	0.934
Subclinical Hypothyroidism	30 (2.54%)	1,096 (2.36%)	1.24	0.76–2.01	0.394
Hypothyroxinemia	30 (2.54%)	1,011 (2.18%)	1.02	0.68–1.55	0.910

Odds ratios (OR) are adjusted for maternal age, pre-pregnancy BMI, education level, TPOAb status, and IVF pregnancy. Diagnostic criteria for thyroid disorders were based on twin-specific reference ranges for twin pregnancies and singleton-specific reference ranges for singleton pregnancies.

In this study, three different thyroid function reference ranges were applied to assess abnormalities in TSH and FT4 levels during the first trimester in women with twin pregnancies: a twin pregnancy-specific reference range, a singleton pregnancy-specific reference range, and the ATA-recommended reference range. Based on each reference standard, we identified cases of high or low TSH/FT4 and evaluated their associations with adverse pregnancy outcomes, including preterm birth, preeclampsia, GDM, SGA, LGA, fetal distress, and emergency cesarean section. We used univariate logistic regression models to calculate the proportion of abnormal thyroid function, the incidence of each adverse outcome, the ORs and corresponding 95% CI under each reference standard. In addition, the sensitivity, specificity, positive predictive value (PPV), and negative predictive value (NPV) of each reference range were evaluated. A P-value < 0.05 was considered statistically significant. Our results showed that, in predicting pregnancy complications, low TSH defined by the twin-specific reference range was significantly associated with preeclampsia (OR = 2.59, 95% CI: 1.18–5.71, P = 0.014). Under the similar NPV, this reference showed a higher specificity (97.6%) and a higher PPV (28.1%) compared to the other standards. both superior to the singleton-specific and ATA reference ranges. In contrast, low TSH defined by the singleton-specific and ATA standards was not significantly associated with preeclampsia (P = 0.788 and 0.140, respectively). In terms of fetal outcomes, low FT4 defined by the twin-specific range was significantly associated with SGA (OR = 2.52, 95% CI: 1.14–5.61, P = 0.019), LGA (OR = 6.13, 95% CI: 1.51–24.9, P = 0.011), emergency cesarean section (OR = 2.68, 95% CI: 1.00–7.19, P = 0.042), and fetal distress (OR = 2.64, 95% CI: 1.15–6.05, P = 0.020). These associations were accompanied by consistently high specificity and relatively high PPV, while maintaining comparable NPV. By comparison, under the singleton-specific standard, only the associations with LGA and fetal distress reached statistical significance, and both specificity and PPV were slightly lower. Detailed results are shown in **Table 4**. In summary, the twin-specific reference range demonstrated superior accuracy in identifying truly abnormal and high-risk cases among women with twin pregnancies. It significantly reduced misclassification and overdiagnosis while maintaining high confidence in negative results.

**Table 4:** Diagnostic performance of different thyroid function reference ranges in predicting adverse pregnancy outcomes.

Adverse outcome	Indicator	Reference criteria	Abnormal cases (%)	Outcome rate (%)	OR (95% CI)	P-value	Sensitivity (%)	Specificity (%)	PPV (%)	NPV (%)
Preeclampsia	Low TSH	Twin-specific	32 (2.7%)	28.1%	2.59 (1.18–5.71)	0.014*	5.7	97.6	28.1	86.9
		Singleton-specific	182 (15.4%)	17.6%	1.11 (0.52–2.40)	0.788	20.4	85.4	17.6	87.4
		ATA criteria	282 (23.8%)	16.0%	1.33 (0.91–1.93)	0.140	28.7	76.8	16.0	87.5
SGA	Low FT4	Twin-specific	30 (2.5%)	30.0%	2.52 (1.14–5.61)	0.019*	5.2	97.7	30.0	85.0
		Singleton-specific	58 (4.9%)	17.2%	1.25 (0.62–2.53)	0.536	6.6	94.9	17.2	85.7
LGA	Low FT4	Twin-specific	2.6%	10.0%	6.13 (1.51–24.9)	0.011*	13.0	97.6	10.0	98.2
		Singleton-specific	5.52%	6.9%	4.01 (1.34–12.1)	0.009*	18.2	94.7	6.9	98.2
Emergency CS	Low FT4	Twin-specific	30 (2.5%)	16.7%	2.68 (1.00–7.19)	0.042*	6.0	97.3	16.7	92.9
		Singleton-specific	58 (4.9%)	12.1%	1.81 (0.79–4.14)	0.154	8.3	94.9	12.1	92.4
Fetal Distress	Low FT4	Twin-specific	2.6%	26.7%	2.64 (1.15–6.05)	0.020*	5.6	97.8	26.7	87.8
		Singleton-specific	5.52%	22.4%	2.12 (1.06–4.22)	0.028*	9.8	95.1	22.4	88.0

*P-values < 0.05 are considered statistically significant.

PPV, Positive Predictive Value; NPV, Negative Predictive Value; OR, Odds Ratio; CI, Confidence Interval; SGA, Small for Gestational Age; LGA, Large for Gestational Age; CS, Cesarean Section.

Abnormal thyroid function is defined based on the respective reference criteria.

*P-values < 0.05 are considered statistically significant, and bold values indicate significance.

### Univariate and multivariate analyses of thyroid function abnormalities and pregnancy outcomes

**Figure 4:**
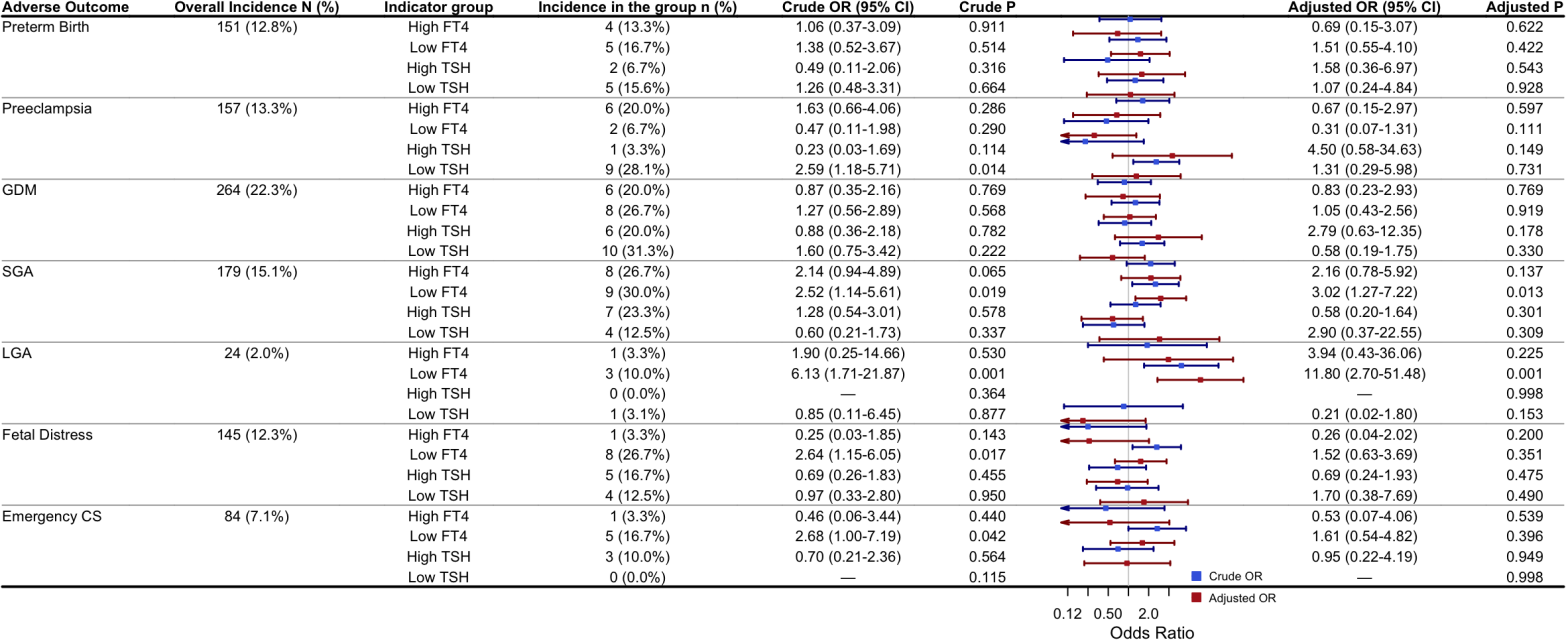
Univariate and multivariate logistic regression analyses of the association between abnormal FT4/TSH and adverse pregnancy outcomes: Adjusted ORs were obtained from multivariable logistic regression models adjusting for maternal age, pre-pregnancy BMI, education level, TPOAb status, and use of IVF. High or low FT4/TSH were defined as >97.5th or <2.5th percentile based on cohort-specific reference ranges for twin pregnancies. The reference group for the regression analyses was participants with FT4 and TSH levels within the normal range. P-values < 0.05 were considered statistically significant. Abbreviations: GDM, Gestational diabetes mellitus; SGA, Small for gestational age; LGA, Large for gestational age; IVF, *in vitro* fertilization; CS, Cesarean section.

To further investigate the associations between thyroid function abnormalities and adverse pregnancy outcomes, both univariate and multivariate logistic regression analyses were performed to assess the relationships between abnormal FT4 or TSH levels and the outcomes mentioned above. In the multivariate logistic regression model, potential confounders (including maternal age, pre-pregnancy BMI, educational level, TPOAb status, and IVF use) were adjusted for. Adjusted odds ratios (aORs) and their 95% CIs were calculated. The results are presented in **Figure 4**. Univariate logistic regression analysis showed that low FT4 was significantly associated with an increased risk of GDM, SGA, LGA, and fetal distress (all P < 0.05), while low TSH was significantly associated with preeclampsia (P = 0.014). After adjusting for the potential confounders, multivariate logistic regression revealed that only low FT4 remained significantly associated with the risks of SGA (aOR = 3.02, 95% CI: 1.27–7.22, P = 0.013) and LGA (aOR = 11.80, 95% CI: 2.70–51.48, P = 0.001). No statistically significant associations were observed between abnormal FT4 or TSH levels and other adverse pregnancy outcomes, including preterm birth, preeclampsia, GDM, fetal distress, or emergency cesarean section (all P > 0.05).

### Effect modification analysis of Low FT4 associations with SGA and LGA by maternal characteristics

To explore whether the association between low FT4 and the risks of SGA and LGA varies by maternal characteristics, stratified and interaction analyses were conducted. The results showed no statistically significant interactions between FT4 levels and all tested maternal variables, including pre-pregnancy BMI, maternal age, educational level, TPOAb status, and IVF use (**Table 5**). This suggests that the effect of low FT4 on the risks of SGA and LGA may be relatively consistent across the study population, with no evident heterogeneity observed. However, the limited number of events in some subgroups, particularly in the LGA analysis, may have reduced the statistical power to detect interactions, highlighting the need for further studies with larger sample sizes.

**Table 5:** Stratified comparison of the incidence of SGA and LGA in the low FT4 group and the corresponding adjusted odds ratios relative to the normal FT4 group.

Variable	Group (%)	SGA					LGA				
		No (%)	Yes (%)	aOR (95% CI)	P-value	P for interaction	No (%)	Yes (%)	aOR (95% CI)	P-value	P for interaction
All participants	1153 (100.0%)	2.1%	5.3%	2.85 (1.19–6.82)	0.018*	–	2.4%	13.0%	13.71 (3.19–58.89)	<0.001*	–
BMI ≥ 24 kg/m²						0.458					0.986
No	611 (80.5%)	3.1%	6.0%	2.51 (0.93–6.79)	0.070		3.2%	30.0%	19.74 (4.15–93.81)	<0.001*	
Yes	148 (19.5%)	3.7%	15.4%	4.33 (0.68–27.67)	0.121		4.8%	0.0%	–	0.998	
Maternal age ≥ 35y						0.137					0.988
No	919 (79.7%)	1.4%	5.6%	4.49 (1.63–12.37)	0.004*		1.8%	15.8%	22.73 (4.45–116.04)	<0.001*	
Yes	234 (20.3%)	4.9%	3.4%	0.67 (0.08–5.81)	0.718		4.8%	0.0%	–	1.000	
Bachelor’s degree or higher						0.988					0.847
No	377 (32.7%)	1.2%	0.0%	–	0.997		0.8%	7.1%	29.86 (1.35–659.32)	0.031*	
Yes	776 (67.3%)	2.6%	7.6%	3.71 (1.49–9.21)	0.005*		3.0%	22.2%	14.84 (2.36–93.46)	0.004*	
TPOAb status						0.986					0.988
Negative	1050 (91.1%)	2.1%	5.8%	3.13 (1.29–7.60)	0.012*		2.4%	14.3%	19.09 (4.10–88.94)	<0.001*	
Positive	103 (8.9%)	2.3%	0.0%	–	0.999		2.0%	0.0%	–	1.000	
IVF pregnancy						0.420					0.620
No	521 (45.2%)	0.7%	4.3%	4.62 (0.88–24.37)	0.071		1.2%	9.1%	–	0.995	
Yes	632 (54.8%)	3.3%	6.3%	2.25 (0.78–6.49)	0.135		3.3%	16.7%	11.59 (1.98–67.96)	0.007*	

“No (%)” and “Yes (%)” refer to the absence or presence of SGA/LGA within the low FT4 group. Adjusted odds ratios (aOR) are shown for the risk of SGA or LGA in the low FT4 group compared with the normal FT4 group. aORs and 95% CIs were derived from stratified multivariable logistic regression models. All models were adjusted for maternal age, pre-pregnancy BMI, education level, TPOAb status, and IVF pregnancy. The reference group in this table is the normal FT4 group (P2.5 - P97.5). *P-values < 0.05 were considered statistically significant. Abbreviations: SGA, Small for gestational age; LGA, Large for gestational age; IVF, *in vitro* fertilization; BMI, body mass index.

*P-values < 0.05 are considered statistically significant, and bold values indicate significance.

### Validity of the 2.5th percentile cutoff for FT4 in identifying SGA and LGA

Currently, the reference interval from the P2.5 to P97.5 is widely adopted internationally as the normal range for thyroid hormones. This statistical approach, based on the distribution of hormone levels in healthy populations, is broadly recognized by clinical laboratories and epidemiological studies, and the 2017 ATA guidelines also recommend it as the reference range for thyroid hormones (9). However, this method has inherent limitations. Firstly, it assumes a normal or near-normal distribution of hormone levels in healthy individuals and defines the “common value range” rather than a clinically “risk-free” threshold. Secondly, it arbitrarily sets 5% of the population as abnormal, which may result in some individuals with borderline hormone levels but potential clinical risks being classified as “normal.” Lastly, the reference interval is not equivalent to diagnostic criteria; some pregnant women with thyroid dysfunction may have hormone levels within the statistical “normal” range yet still experience adverse pregnancy outcomes.

Against this background, our study further explored the impact of FT4 levels in the borderline low range (P2.5–P5) on the risks of LGA and SGA, aiming to evaluate the capability of the current reference interval in identifying clinically relevant borderline risk groups (**Table 6**). Participants were divided into three groups based on FT4 levels: the extreme group (FT4 < P2.5), the marginal group (FT4 between P2.5 and P5), and the intermediate normal group (FT4 between P5 and P95).

**Table 6:** Association between low FT4 levels and risks of LGA and SGA using intermediate normal group as reference.

Outcome	Group comparison	Incidence rate (%)	Crude OR (95% CI)	Crude P	Adjusted OR (95% CI)	Adjusted P
LGA	Marginal group (P2.5–P5)	3.23%	1.93 (0.25–14.96)	0.528	2.03 (0.23–17.65)	0.522
	Extreme group (<P2.5)	10.0%	6.44 (1.79–23.19)	0.004*	13.95 (3.21–60.64)	<0.001*
SGA	Marginal group (P2.5–P5)	3.2%	0.20 (0.03–1.44)	0.109	0.24 (0.03–1.79)	0.164
	Extreme group (<P2.5)	30.0%	2.51 (1.13–5.58)	0.024*	2.77 (1.16–6.64)	0.022*

*Adjusted odds ratios (aOR) were derived from multivariable logistic regression models adjusting for maternal age, BMI, education level, TPOAb status, and IVF conception. The reference group for all comparisons was the Intermediate normal group (FT4 between P5 and P95).

*P-values < 0.05 are considered statistically significant, and bold values indicate significance.

The incidence rates of LGA were 10.0%, 3.23%, and 1.7% across the extreme group, marginal group, and intermediate normal group, respectively. After multivariate adjustment, the extreme group had a significantly increased risk of LGA (aOR = 13.95, 95% CI: 3.21–60.64, P < 0.001); although the marginal group showed an elevated trend (aOR = 2.03), it was not statistically significant (P = 0.522). Similarly, the incidence rates of SGA were 30.0%, 3.2%, and 14.6% across the three groups, respectively. Multivariate analysis demonstrated a significantly increased risk of SGA in the extreme group (aOR = 2.77, 95% CI: 1.16–6.64, P = 0.022); the marginal group, however, showed a non-significant downward trend (aOR = 0.24, 95% CI: 0.03–1.79, P = 0.164). These findings further support the scientific and clinical validity of using P2.5 as the lower limit of FT4 abnormality, as this threshold effectively identifies high-risk pregnant women with adverse outcomes. The marginal range (P2.5–P5) did not show a significant risk in this study, suggesting no current need to adjust the existing reference interval. The current reference range demonstrates good risk identification capability in clinical practice.

## Discussion

### Main findings

This study established pregnancy-specific reference ranges for early pregnancy TSH and FT4 based on large cohorts of singleton (n = 46,474) and twin (n = 1,183) pregnancies. Compared to singleton pregnancies, twin pregnancies showed significantly lower TSH levels in early pregnancy, with a downward shift in the reference range. In contrast, FT4 levels were higher, and the reference range was broader, suggesting a distinct thyroid function profile in twin pregnancies. These findings suggest that thyroid function in twin pregnancies is more dynamic, likely due to an adaptive response driven by elevated hCG levels and increased metabolic demands. The broader FT4 reference range may reflect greater physiological variation in thyroid hormone regulation among individuals during twin pregnancies. This also underscores the importance of developing twin-specific thyroid function reference ranges. Further comparison of diagnostic criteria for thyroid dysfunction in twin pregnancies revealed that using twin-specific reference values significantly reduced the detection rates of abnormal TSH and FT4 levels compared to the ATA guidelines and local singleton reference ranges. This suggests that applying non–twin-specific reference intervals may overestimate abnormality rates, potentially leading to unnecessary clinical interventions. Univariate analyses exploring the association between early pregnancy thyroid dysfunction and adverse pregnancy outcomes showed that twin-specific low TSH was notably associated with an increased risk of preeclampsia, whereas low FT4 was associated with adverse fetal outcomes such as SGA, LGA, emergency cesarean delivery, and fetal distress. Compared to singleton and ATA standards, the twin-specific criteria demonstrated higher specificity and PPV, indicating their effectiveness in identifying truly high-risk individuals. This suggests that twin-specific reference ranges could serve as valuable tools for precision pregnancy management, especially in the context of twin pregnancies. Building on these findings, multivariate regression analyses adjusting for maternal age, pre-pregnancy BMI, education level, TPOAb status, and IVF use confirmed that low FT4 remained significantly associated with increased risks of SGA and LGA. This highlights the close relationship between decreased FT4 levels and fetal growth abnormalities in twin pregnancies, underscoring the need to focus on FT4 monitoring and tailored interventions in clinical management for this population. Further stratified analyses showed that the associations between low FT4 and risks of SGA and LGA were consistent across different maternal subgroups, with no significant interaction effects. This suggests a certain degree of generalizability of these findings across diverse populations; however, larger studies are needed to validate the stability and broader applicability of these associations. To further assess the ability of current reference intervals to identify borderline risk groups, we examined the impact of FT4 levels in the marginally low range (P2.5–P5) on risks of LGA and SGA. Results showed no significant risk increase in this marginal group, providing no support for adjusting the existing reference ranges. Overall, the current reference intervals demonstrate good performance in risk identification.

### The clinical importance of twin-specific thyroid reference ranges in early pregnancy

For many years, it has been recognized that thyroid function during pregnancy is critical to maternal and fetal health. Maternal thyroid hormones may influence placental development, and the transfer of maternal T4 across the placenta can affect normal fetal development (11). Studies have linked thyroid dysfunction to risks such as preterm birth, miscarriage, and impaired fetal neurodevelopment. Therefore, some experts advocate for treatment in these cases. However, the risks of overtreatment have also been documented. Unnecessary therapy, particularly thyroid hormone supplementation, can expose both mother and fetus to potential side effects (2). Insufficient or excessive treatment of thyroid disorders, inappropriate choice of therapeutic regimen, or delays in initiating or adjusting treatment may all adversely affect pregnancy and fetal outcomes (11). For instance, research shows a U-shaped relationship between maternal FT4 levels and child IQ, gray matter volume, and cortical brain volume, with high FT4 also associated with increased incidence of attention deficit hyperactivity disorder (ADHD) and behavioral problems (12, 13). Reported prevalence of thyroid disorders during pregnancy varies across studies, largely due to differences in reference ranges used (14). Physiological changes in thyroid function and hormone metabolism during pregnancy have led many researchers to recommend pregnancy- and population-specific TSH and thyroid hormone reference intervals. Factors such as BMI, iodine intake, ethnicity, and presence of anti-thyroid antibodies may influence reference values (14, 15). Consequently, unified reference ranges recommended by endocrine societies may misclassify healthy pregnant women as abnormal (16). Accurate diagnosis requires reliable, individualized reference intervals (17). Although scientific associations are developing population-specific thyroid function reference ranges (17, 18), twin pregnancies are often excluded due to their unique physiological differences (19), including higher hCG and estrogen levels (20). In early twin pregnancies, FT4 is elevated due to hCG stimulation (3), with both total and free β-hCG peaking significantly higher and lasting longer between 8 and 16 weeks gestation. Serum hCG increases by 10,000 U/L typically lower serum TSH by 0.1 mU/L, reflecting the inverse relationship throughout pregnancy, which may cause more pronounced physiological TSH suppression in twin pregnancies (21). Moreover, increased metabolic demands in twin pregnancies raise the risk of various nutritional deficiencies that can affect thyroid hormone secretion (22). Previous attempts to establish twin-specific reference ranges have been limited by varying assay methods, making direct comparison difficult (23). Our study found that early-pregnancy TSH median and reference range were significantly lower in twins compared to singletons, while FT4 median and reference range were significantly higher. These findings reflect the unique thyroid function regulation in twin pregnancies. Using singleton or guideline reference ranges directly may lead to misclassification of thyroid dysfunction, underscoring the importance of twin-specific reference intervals. When compared with universal standards, applying ATA guidelines or singleton reference ranges in early pregnancy significantly overestimated the prevalence of thyroid dysfunction in twin pregnancies. Using twin-specific reference intervals brought detection rates closer to those of singletons. This emphasizes the need for dedicated twin pregnancy reference ranges to avoid unnecessary clinical intervention. While twin pregnancies have been thought to carry a higher risk of thyroid dysfunction, our findings suggest this difference may mainly stem from the inappropriate use of singleton reference intervals, leading to overdiagnosis and potentially unnecessary treatments, wasted healthcare resources, and increased psychological burden on pregnant women.

### Incidence of thyroid dysfunction subtypes in twin pregnancies

When analyzing specific subtypes, the prevalence of overt hyperthyroidism diagnosed using the twin-specific criteria was slightly lower, consistent with findings from another study in China (24). That study reported an overt hyperthyroidism rate of 0.9% in early pregnancy based on singleton criteria, which dropped to 0% when twin-specific standards were applied. This suggests that applying singleton reference ranges to twin pregnancies may misclassify normal physiological changes as hyperthyroidism. This conclusion aligns with physiological mechanisms: despite higher hCG levels in twin pregnancies, thyroid receptor sensitivity decreases due to the “plateau effect” of hCG, resulting in a low true incidence of overt hyperthyroidism. Additionally, twin pregnancies tend to begin prenatal care earlier and have more frequent monitoring, which may further reduce the occurrence of overt hyperthyroidism.

Second, no cases of overt hypothyroidism were detected in the twin pregnancy group in this study, though this finding should be interpreted with caution. Elevated hCG levels in early twin pregnancies lead to increased FT4 and suppressed TSH, representing a state of compensated thyroid hyperfunction that may mask underlying hypothyroidism. Considering that the prevalence of overt hypothyroidism in singleton pregnancies is approximately 0.2 ~ 0.5% (25, 26), the absence of overt hypothyroidism in this cohort is consistent with this physiological mechanism. However, due to the limited sample size, future studies with larger populations are warranted to further evaluate this risk. Moreover, the incidence of subclinical hyperthyroidism was slightly higher among twin pregnancies, which aligns with theoretical expectations. The higher and more prolonged hCG peak in twin pregnancies tends to suppress TSH levels, making subclinical hyperthyroidism more common; this condition is usually physiological and does not require treatment. The rates of subclinical hypothyroidism and hypothyroxinemia were also slightly increased, consistent with the heightened thyroid hormone demand in twin pregnancies. In summary, based on these findings, twin-specific reference intervals should be used when screening thyroid function in twin pregnancies to improve diagnostic accuracy and avoid overdiagnosis and unnecessary therapeutic interventions.

### Twin-specific reference ranges for thyroid dysfunction in predicting adverse pregnancy outcomes

In this study, further evaluation of the association between thyroid dysfunction subtypes and adverse pregnancy outcomes revealed that twin-specific reference ranges offer a stricter and more accurate classification of euthyroid pregnant women. This significantly reduces false-positive cases, meaning fewer healthy women are misdiagnosed with thyroid dysfunction. The increase in PPV indicates that when thyroid function tests are flagged as abnormal, they are more likely to be truly linked with adverse pregnancy outcomes, thereby enhancing the reliability and clinical significance of such diagnoses. Conversely, the NPV remained stable, suggesting that the ability of this standard to identify low-risk women and exclude normal thyroid function is not compromised. Although the twin-specific reference ranges led to marked improvements in specificity and PPV for predicting adverse outcomes—with NPV also maintained—sensitivity decreased. This change largely results from the stricter diagnostic thresholds, which effectively reduce false positives and improve the accuracy and clinical relevance of the results. However, this also means some high-risk women with borderline thyroid function abnormalities may be missed, resulting in lower sensitivity. From an etiological standpoint, adverse pregnancy outcomes such as preeclampsia and preterm birth are complex and multifactorial, with thyroid dysfunction being only one contributing risk factor. Therefore, even pregnant women whose thyroid function falls within the twin-specific “normal” range may still experience adverse outcomes due to other factors. In other words, the reduction in sensitivity reflects the complex causes of these outcomes and underscores the limitations of relying solely on thyroid function markers for prediction. Hence, establishing pregnancy type- and gestational age-specific thyroid function reference ranges is crucial for achieving precise diagnosis, avoiding overtreatment, and optimizing healthcare resource allocation.

Notably, our study found that lower FT4 levels in twin pregnancies may have a dual effect on fetal growth, increasing the risk of both SGA and LGA. This finding helps explain seemingly conflicting reports in the literature, suggesting that the relationship is not contradictory but rather reflects different mechanisms through which low FT4 influences pregnancy outcomes. Some studies support the link between low FT4 and SGA. For example, a Japanese study reported that women with isolated hypothyroxinemia or subclinical hypothyroidism in early pregnancy had a higher likelihood of delivering SGA infants (27). A meta-analysis also showed that levothyroxine (L-T4) treatment in pregnant women with subclinical hypothyroidism reduced the risks of preterm birth and low birth weight (28). Mechanistically, thyroid hormone deficiency may lead to endothelial dysfunction (29), reduce the production of vasodilators such as nitric oxide (30), and impair placental blood flow. In addition, placental development relies on thyroid hormone-regulated cell migration and blood vessel formation. Low FT4 may weaken the anti-inflammatory environment of the placenta, disrupt its normal development, and contribute to fetal growth restriction (31). On the other hand, evidence also links low FT4 with LGA. Li et al. found that isolated maternal hypothyroxinemia in early pregnancy was associated only with LGA (32). Another study observed that maternal FT4 levels in early pregnancy were negatively correlated with neonatal birth weight, and the incidence of LGA was increased among male infants born to mothers with subclinical hypothyroidism (33). Cleary-Goldman et al. also reported that low thyroid hormone levels in early pregnancy were related to macrosomia (34). Previous research from our institution similarly showed that lower FT4 in early pregnancy was associated with higher birth weight and an increased risk of LGA (35). These findings suggest alternative pathways: for example, low FT4 may raise blood glucose levels and enhance placental glucose transport, promoting fetal overgrowth (36). Another hypothesis is that hypothyroidism may contribute to maternal lipid abnormalities, such as high cholesterol, elevated low-density lipoprotein (LDL), and increased apolipoprotein B (apo B) (37), which can negatively affect birth weight. In summary, the observed dual increase in SGA and LGA risks associated with low FT4 suggests that FT4 may act as a common upstream factor, influencing fetal growth through different pathways, by impairing placental structure and function on one hand, and disrupting maternal metabolic homeostasis on the other. This highlights the complex role of FT4 in fetal growth and underscores the need for further research into how FT4 affects placental function, nutrient transport, and fetal metabolism. In contrast, abnormal TSH levels did not show statistically significant associations with fetal growth, consistent with previous twin pregnancy studies (38). This may be explained by the immature hypothalamic-pituitary-thyroid (HPT) axis in the fetus before 18–20 weeks of gestation, during which fetal TSH levels remain very low and pituitary feedback is not fully developed, resulting in weaker FT4 suppression of TSH. The HPT axis only fully matures 1~2 months after birth, partially explaining the lack of correlation between TSH and birth weight. Furthermore, FT4, as a direct marker of thyroid function, may more sensitively reflect the potential influence of thyroid hormones on pregnancy outcomes than TSH. Given the relatively low incidence of LGA in twin pregnancies, the absolute number of cases in this study was limited, which may affect statistical power. Therefore, the observed association between decreased FT4 and increased LGA risk should be interpreted cautiously and warrants confirmation in larger cohorts.

### Thyroid function screening in asymptomatic pregnant women

Currently, there is no consensus among international societies regarding universal thyroid function screening in asymptomatic pregnant women. The ATA guidelines indicate that evidence is insufficient to recommend or oppose universal TSH screening in early pregnancy and do not recommend routine FT4 testing, suggesting a case-finding approach for high-risk women (9). Similarly, the RCOG guideline (11), the Endocrine Society (39) and the American Society for Reproductive Medicine (ASRM) (40) recommend screening only in high-risk populations rather than universally, with TSH measurement reserved for women with clear risk factors. However, some studies have suggested that universal screening may be cost-effective (41, 42). Considering China’s healthcare resources and cost-effectiveness, the Chinese Guidelines for the Diagnosis and Treatment of Thyroid Disease during Pregnancy and Postpartum support early pregnancy thyroid function screening in hospitals and maternal-child health institutions where resources allow, including serum TSH, FT4, and TPOAb (10). This strategy aims to facilitate early detection and management of thyroid disorders, particularly in high-risk populations such as twin pregnancies. Further research is needed to evaluate the effectiveness and cost-effectiveness of universal screening across different populations and resource settings.

### Strengths and limitations

This study is one of the few large-sample investigations in the Chinese population to establish twin pregnancy-specific thyroid function reference intervals. We developed early pregnancy-specific reference ranges for TSH and FT4 for both singleton and twin pregnancies, fully accounting for the unique physiological changes seen in twin gestations. This work addresses the current gap in related research within the Chinese context and provides localized data to support the refined management of multiple pregnancies. By comparing the ATA guidelines, local singleton standards, and twin-specific reference ranges, we demonstrated that using twin-specific intervals significantly reduces the overdiagnosis of thyroid dysfunction and unnecessary clinical interventions, highlighting their practical clinical value. Additionally, the use of multivariate regression analyses to adjust for multiple confounding factors enhances the reliability and scientific rigor of our findings. Stratified analyses further confirmed the generalizability and stability of the results. Lastly, the detailed examination of borderline risk ranges provides empirical support for the appropriateness of the proposed reference intervals.

However, several limitations should be noted. First, although the sample size is substantial, the data were collected from a single center and a specific population, which may limit the generalizability and representativeness of the findings. Multi-center and multi-regional studies are needed to validate these results further. Second, as a retrospective study, despite adjusting for some confounders in multivariate analysis, certain potential influencing factors such as chorionicity in twin pregnancies, serum hCG levels, and iodine nutritional status could not be fully accounted for. Although regional monitoring data in Shanghai indicate that, since the implementation of universal salt iodization in 1996, iodine nutrition in the local population has generally remained adequate and safe, with studies suggesting sufficient iodine intake among children and pregnant women in the area (43), the lack of individual-level iodine measurements may introduce residual confounding in interpreting thyroid function indicators. Therefore, causal inferences should be made with caution. Moreover, the study did not include continuous monitoring of thyroid function throughout pregnancy, limiting insights into dynamic changes over the course of gestation. Future research should incorporate multicenter data and longitudinal designs to further refine and validate these findings. Finally, due to the limited number of twin pregnancies with concurrent abnormal FT4 and TSH in this study, we did not perform a combined analysis of their impact on obstetric and fetal outcomes. Future studies with larger twin cohorts are needed to explore this joint effect.

### Future perspectives

Building upon the findings and limitations of this study, future research should consider the following directions: First, large-scale, multicenter prospective studies are needed to validate the applicability and consistency of twin-specific thyroid function reference ranges across different populations and geographic regions, ultimately contributing to the establishment of more universally accepted standards. Second, further investigation into the interactions between key biomarkers such as hCG and placental hormones and dynamic changes in thyroid function is essential to better understand the unique endocrine environment in twin pregnancies. Third, by integrating maternal characteristics, hormone profiles, and thyroid function parameters, predictive models could be developed to assess the risk of adverse pregnancy outcomes in twin gestations. Such models would support early risk stratification and individualized clinical management. Finally, intervention trials based on the current findings are warranted to evaluate whether correcting thyroid dysfunction particularly low FT4 can improve pregnancy outcomes. These studies would provide higher-level evidence to inform precision management strategies for twin pregnancies.

## Data Availability

The raw data supporting the conclusions of this article will be made available by the authors, without undue reservation.
